# Editorial: Persistent Activity in the Brain – Functions and Origin

**DOI:** 10.3389/fncir.2021.841451

**Published:** 2022-02-08

**Authors:** Shintaro Funahashi, Emmanuel Procyk

**Affiliations:** ^1^Advanced Research Institute for Multidisciplinary Science, Beijing Institute of Technology, Beijing, China; ^2^Shenzhen Institute of Advanced Technology, Chinese Academy of Science, Shenzhen, China; ^3^Univ Lyon, Université Lyon 1, Inserm, Stem Cell and Brain Research Institute U1208, Bron, France

**Keywords:** prefrontal cortex, dynamic code, working memory, network, model

Since Fuster and Alexander first found persistent firing in the prefrontal cortex during delayed-response performance (Fuster and Alexander, 1971), persistent neuronal activity has been observed in multiple brain areas and species, in relation to a variety of cognitive activities that require sustained processing or maintenance of states to bridge different events separated in time or to control or monitor specific functions. Persistent delay-period activity observed in the prefrontal cortex has been shown to play important roles to temporarily maintain either retrospective or prospective information necessary to perform a variety of cognitive behaviors during the delay interval, which corresponds to the function of working memory. Therefore, persistent activity has been considered as a neural correlate of working memory. Although it is an important phenomenon to understand neural mechanisms of working memory, planning or cognitive control, debates have been continued among researchers regarding its meaning, sources, and even relevance. In this Research Topic, we offered an opportunity to present research outputs carried out with various approaches including electrophysiology, behavior, and computational modeling, and to further discuss the origin and functional significance of persistent activity.

The brain uses different timescales to process information. Cavanagh et al. explored the features of persistent activity based on intrinsic timescales in single neurons for information processing. As primary sensory areas process momentary sensory inputs regardless of their context, short neural timescales in turn contribute to processing and representing such information. However, neural activity in association areas that integrate various information to achieve distant goals, are characterized by longer timescale. Single neurons display different individual timescales, which predict the strength of mnemonic encoding. Neurons exhibiting longer timescales play greater roles for stable maintenance of mnemonic information and for integrating multiple pieces of task-relevant information. Although neurons exhibiting shorter timescales are also present within higher cortical areas, the presence of neurons exhibiting heterogeneous timescales could be important for adapting environmental demands dynamically.

Although persistent activity has been frequently observed in the prefrontal cortex, this activity has also been observed in many other brain areas and the function and the information represented by this activity are not always the same across different brain areas, because different brain areas participate in different information processing. Roussy et al. dissociated neural processes for visual perception from those of visual working memory based on whether the brain produces mental representations of the visual stimulus when its physical signals are available or not. In fact, neurons in early visual areas exhibit persistent activity representing perceptual signals, while neurons in association areas exhibit the activity representing working memory signals. Persistent activity has also been observed in human brain imaging studies, although not in all frontal regions observed in monkey studies. Curtis and Sprague reviewed these results obtained with fMRI signals, and showed that the content of working memory can be decoded from the pattern of neural activations in several brain areas, suggesting that these approaches find which of these brain areas contribute to working memory and what relevant features they represent.

Li et al. showed that persistent activity is observed even in naïve untrained monkeys, indicating that specific local neural circuits supporting persistent activity are innately present in the prefrontal cortex. However, the operation of these circuits is flexibly modulated by the task demand, the experience, and the age and also by the effect of neuromodulators. Systemic muscarinic blockade disrupts working memory performances. Vijayraghavan and Everling reviewed such neuromodulatory effects of acetylcholine on persistent activity in the prefrontal cortex. They showed that muscarinic blockade by local iontophoretic application caused pronounced suppressive effect of persistent activity representing remembered information and that the suppressive effect of persistent activity is dose-dependent and monotonically through muscarinic M1 receptors. The observed heterogeneity of muscarinic actions also outlines unexpected modulatory effects in primate prefrontal cortex when compared with rodent studies.

Recurrent neural circuit models have been proposed to explain how sustained representations are generated. Barbosa et al., Novikov et al., and Sarazin et al. have used such models of uni—or—multi-area networks to examine features of persistent or dynamic activity. Models are often informed by known biological elements and properties of recurrent circuits reviewed by Li et al., Curtis and Sprague, Roussy et al., Sarazin et al. used a recurrent neural circuit model with spike timing-dependent synaptic plasticity and showed that this model can learn, memorize, and replay a large-size of continuous dynamical sequences of spiking activity under asynchronous irregular nature at different timescales, which replicates the dual dynamical and persistent aspects of working memory representations observed in the prefrontal cortex.

Although persistent activity has been observed in single-neuron studies while monkeys performed delay tasks, such activity is often unstable along the delay period and heterogeneous within and across trials, which raises questions on how it contributes to working memory or to other processes putatively supported by tonic neural processing. In fact, dynamic coding and multiplexing during memory delays have been observed and are proposed to maximize the dimensionality of neural representations (Amengual and Ben Hamed; Cavanagh et al.; Curtis and Sprague; Sarazin et al.). Amengual and Ben Hamed suggested that heterogeneous features of persistent activity are caused by intrinsic oscillatory dynamics working at multiple timescales and that these features allow to dynamically incorporate multiple sources of information.

When subjects perform working memory tasks, an increase of gamma-band activity has been observed and is considered to reflect activation of neural populations representing the content of working memory. Novikov et al. examined functions of gamma-band oscillation on persistent activity by investigating joint effects of gamma-band oscillatory inputs and noise on the dynamics of the neural circuit with a metastable active condition. They showed that gamma-band oscillations are able to preferentially stabilize the active condition of the circuit in which information is retained in working memory, and that the synchronization of gamma oscillators affects the ability of the gamma inputs to stabilize the retention of working memory, indicating an importance of gamma-band oscillation for maintaining information in working memory. Importance of gamma-band oscillation is also shown for maintaining multiple items in working memory simultaneously. Barbosa et al. hypothesized that different features of an object (e.g., color and position) stored in different cortical areas are bound in memory through synchrony across feature-specific neural populations and tested this hypothesis using a neural network model composed of two one-dimensional attractor networks (one for color and one for position). They found that different memorized items are held at different phases of the network oscillation, that binding is accomplished through the synchronization of parts of bumps across the brain areas, and that encoding and decoding of object features are accomplished through rate coding.

Thus, although the debate regarding the meaning, source and relevance of persistent activity is continued, the maintenance of information and of neural patterns is still a central phenomenon to understand the neural mechanisms of working memory. Further insights might come from understanding whether and how intrinsic oscillatory dynamics working at multiple timescales contribute to generating persistent information. Also, as Curtis and Sprague suggested, decoding information represented in persistent activity observed in various brain areas may help decipher which brain areas are necessary for working memory and which features are represented and maintained in these brain areas. Pushing the line even further, Fuster observed in his opinion paper that “working memory consists in the temporary activation of an updated cortical network of long-term memory for the attainment of an objective,” and conceptualize it as a *cognit*, an operational memory network. The *cognit* is distributed by nature, and thus, a network level approach is crucial to fully comprehend its functioning.

## Author Contributions

All authors listed have made a substantial, direct, and intellectual contribution to the work and approved it for publication.

## Funding

EP was supported by NORAD ANR-19-CE37-0008-01 and performed within the framework of the LABEX CORTEX (ANR-11-LABX-0042) of Université de Lyon. EP is employed by the Centre National de la Recherche Scientifique.

## Conflict of Interest

The authors declare that the research was conducted in the absence of any commercial or financial relationships that could be construed as a potential conflict of interest.

## Publisher's Note

All claims expressed in this article are solely those of the authors and do not necessarily represent those of their affiliated organizations, or those of the publisher, the editors and the reviewers. Any product that may be evaluated in this article, or claim that may be made by its manufacturer, is not guaranteed or endorsed by the publisher.
